# Management of Acute Uncomplicated Diverticulitis May Exclude Antibiotic Therapy

**DOI:** 10.7759/cureus.1250

**Published:** 2017-05-15

**Authors:** Jonathan Mayl, Mikhail Marchenko, Emily Frierson

**Affiliations:** 1 College of Medicine, University of Central Florida

**Keywords:** diverticulitis, acute uncomplicated diverticulitis, management of diverticulitis, management of acute uncomplicated diverticulitis, antibiotics for diverticulitis, antibiotics, colonic diverticulitis, complications, nonantibiotic management, diverticular disease

## Abstract

Diverticulitis is a common ailment that is prevalent in the developed world. As such, the management of diverticulitis places a substantial economic burden on healthcare. Research is ongoing to further elucidate both the pathogenesis of the disease, as well as ways to reduce associated expenditures. One of these emerging areas of research calls into question the use of antibiotics during treatment of acute uncomplicated diverticulitis. Current guidelines are largely based on expert opinion, with little evidence supporting the standard practice of antibiotic therapy. In this literature review, we have compiled and analyzed the latest collection of evidence in managing acute uncomplicated diverticulitis. There have been two randomized controlled trials (RCTs) performed that assessed the possibility of treating acute uncomplicated diverticulitis without antibiotics. Both the Antibiotika Vid Okomplicerad Divertikulit (AVOD) study and Daniels, et al. have found that an observational approach to acute uncomplicated diverticulitis is not inferior to antibiotic treatment and does not result in increased complication or recurrence rates. We also reviewed a single-center cohort study, a prospective observational study, and two retrospective case-controlled studies comparing observational management versus antibiotic treatment in patients with acute uncomplicated diverticulitis. We found the results were comparable; there was no difference in complication rates or recurrence in any study. The consensus among the studies reviewed challenges the current practice guidelines issued by the American Gastroenterological Association. However, given the geographical difference in diverticular disease and inherent bias found in these studies, we cannot recommend a modification of the guidelines. Based on this literature review, we feel compelled to suggest, and strongly recommend, further research be conducted in the United States in order to bolster the already significant evidence against antibiotic therapy in acute uncomplicated diverticulitis.

## Introduction and background

Diverticular disease places a significant burden on healthcare resources and is responsible for a significant number of gastrointestinal hospital admissions each year. In 2004, 3.2 million people were treated on an outpatient basis and 815,000 people required hospitalization. The pharmacy cost for this care alone totaled $2.8 million [1]. In 2008, the average cost per patient diagnosed with diverticulitis was $9,594. Cumulatively, diverticulitis has placed a $3.066 billion burden on the health care system. This considerable cost is predominantly driven by hospital bed days, as the median length of stay for diverticulitis is three to four days, which accounts for 65-70% of the total healthcare expenditures associated with diverticular disease [1-2]. Although hospital days are the primary driver of healthcare costs, intensive care unit costs, colonoscopies, flexible sigmoidoscopies, computer tomography (CT) scans, ultrasound imaging, clinic and emergency visits, and medications also contribute to the economic impact [3]. 

Colonic diverticula are sac-like protrusions that form at structurally weak points of the colon wall. The presence of diverticula, known as diverticulosis, increases with age. These luminal outpouchings are acquired false diverticula that predominantly occur in the descending and sigmoid colon but are not relegated to these anatomic locations. Their development is largely attributed to the weakening of the colonic wall due to age-related changes, the structure, and motility of the colon. Likely, increased intracolonic pressure from a low fiber diet can exacerbate these weak areas of colonic wall and produce diverticular outpouchings. Among patients with diverticula, 80-85% will remain asymptomatic while approximately 10-25% will experience diverticulitis during their lifetime [4-5]. These paradoxical statistics have led to a deeper investigation of the pathogenesis of diverticular disease. 

Acute diverticulitis is localized inflammation of diverticula and is associated with significant morbidity [6]. While the etiology is not completely understood, diverticulitis may be precipitated by obstruction, stasis, alteration in local bacterial microflora, or ischemia [1]. Newer evidence shows inflammation may play a role in the early pathogenesis. This hypothesis is based on the finding of inflammation found in colonic diverticula without evidence of clinical diverticulitis [7]. The clinical presentation is focal pain and tenderness in the left lower abdominal quadrant, associated with the common laboratory finding of leukocytosis. Other associated symptoms include low-grade fevers, nausea/vomiting, and a change in bowel habits. Approximately 50% of patients report constipation whereas 20-35% of patients report diarrhea in diverticular disease [8]. Abdominopelvic CT scan is considered the gold standard of diagnosis and distinguishes uncomplicated versus complicated diverticulitis [9].

Diverticulitis is divided into two categories: uncomplicated and complicated, the latter defined by complications such as perforations, fissure, obstructions, and bleeding [10]. The medical management of diverticulitis begins with determining the severity of the disease and whether the illness is complicated or uncomplicated [11]. CT scans are instrumental in stratifying patients into either uncomplicated or complicated groups. Current guidelines dictate those who are classified as having a complicated case are hospitalized and have the potential for surgical intervention. However, even patients classified as uncomplicated may require hospitalization if they meet criteria for admission, such as immunosuppression, severe or persistent abdominal pain, intolerance of oral intake, or have significant comorbidities [1]. Those patients who are amenable to outpatient treatment do not meet criteria for hospitalization. Inpatient versus outpatient treatment should be based on a comprehensive assessment of the patient history, physical exam findings, CT scan, laboratory data, and clinical severity [12]. After determining the treatment setting, the choice of pharmacologic intervention and length of treatment needs to be addressed [11].

The common practice consists of antibiotics, bowel rest, and analgesia. Antibiotics are generally prescribed for seven to 10 days with gram-negative and anaerobic coverage [13]. The rationale for antibiotic management is the historically accepted idea that acute diverticulitis is due to an infectious etiology, and treatment with antibiotics would prevent complications [3]. However, the underlying pathophysiology behind diverticulitis is not well understood, and disease resolution without antibiotic treatment has been demonstrated [1-2]. Currently, antibiotics are considered the standard of care for acute uncomplicated diverticulitis (AUD), but the treatment guidelines are based on expert opinion, which is considered class 1C evidence (strong recommendation but low-quality evidence). This has prompted further investigations in an effort to produce recommendations that are better founded on evidence-based medicine [2]. In 2012, the Antibiotika Vid Okomplicerad Divertikulit (AVOD) trial, an RCT, concluded that antibiotic treatment for AUD neither accelerates recovery nor prevents complications or recurrence. These unexpected results have led to a number of studies which further investigated the role of antibiotics in the treatment of AUD. The purpose of this review is to highlight significant findings related to the treatment of diverticulitis in an effort to decrease the significant cost associated with treatment, contribute a stronger evidence-based approach in the treatment of diverticulitis, and attempt to curtail antibiotic resistance related to the overprescribing.

## Review

In 2012, the AVOD group conducted an open, multicenter randomized controlled trial in Sweden. The aim of this study was to investigate the need of antibiotics for treatment of acute uncomplicated left-sided diverticulitis. The study defined uncomplicated diverticulitis as an episode of left lower quadrant clinically consistent with diverticulitis and confirmed by CT. Patients that were diagnosed with diverticulitis but had an abscess, fistula, sepsis, or free air in the abdomen were considered to have complicated diverticulitis. Pain was recorded on a visual analog scale (VAS) and abdominal tenderness at palpation on a scale of 0–4 (tenderness score: 0 - none; 1 - mild local tenderness; 2 - moderate local tenderness; 3 - severe local tenderness; and 4 - local peritonitis).

Patients eligible for the study were randomized into two groups: one that received intravenous fluids only and one that additionally received antibiotics. The antibiotic regime involved initial IV cefuroxime or cefotaxime and metronidazole, or with a carbapenem or piperacillin-tazobactam. This was followed by oral ciprofloxacin or cefadroxil with metronidazole, for a total of at least seven days of antibiotic treatment. Six hundred and sixty-nine patients were randomized: 314 into antibiotics group, 309 into the no-antibiotics group, and 46 were excluded. The median age was 58 and median BMI was 27.7 kg/m2. There were no significant differences between study groups in regards to clinical presentation, including complaints of lower left quadrant pain or presence of fever greater than 100.4 F°. Demographic characteristics and inflammatory parameters were equally balanced between groups. A history of prior diverticulitis was more frequent on the no-antibiotics groups (P = 0.002).

In the primary analysis, there were no significant differences between abdominal pain VAS scores (P = 0.253 - 0.886). Temperatures normalized comparably after two days (P = 0.343) in each group. There was a statistically significant difference on the abdominal tenderness scale during the second day (P = 0.041), with a mean difference from baseline of 0.8 for the no-antibiotics and 1.0 for the antibiotics group. The median hospital stay for both groups was three days. A total of nine patients (1.4%) suffered complications during the initial hospital admission. In the no-antibiotics groups, three patients developed abscesses and three developed perforations, one of which required an emergent sigmoid colectomy. There were three perforations among patients in the antibiotic group, and all three required emergent sigmoid resection. There was no significant difference in complications between groups. Ten patients (3.2%) that were randomized to the no-antibiotics group were treated with antibiotics due to worsening clinical condition, these patients did not develop any further complications during their hospital stay.

In the follow-up analysis, 93 patients (16%) reported recurrent diverticulitis. While no differences were seen between treatment groups, a significant relationship between previous episodes of diverticulitis and recurrence was found (odds ratio: 2.78, 95% confidence interval (CI) 1.76 - 4.41; P = 0.009). There was no difference between surgery at follow-up. Six patients in the no-antibiotics and two patients in the antibiotics group underwent elective or emergent surgery. A total of 41 patients were lost to follow-up. Symptoms of abdominal pain, changes in bowel habit, and extent of diverticulosis at one-year follow-up also did not differ between groups.

The authors concluded that antibiotic therapy does not prevent surgical complications or recurrence. They recommended antibiotics be used mainly for patients with complicated diverticulitis [14].

The AVOD was a randomized, controlled trial conducted without blinding. Allocation and concealment methods were adequate. Study methods and outcome parameters were specific. The attrition rate was acceptable at 13% and patients lost to follow-up and excluded patients were reported. An intention-to-treat analysis was used. Overall, the study appeared well-designed and internally valid. However, the majority of patients in this trial appeared to fit the criteria for outpatient treatment, yet were monitored and treated on an inpatient basis with intravenous antibiotics or fluids. Therefore, the generalizability of these results is somewhat limited.

A 2016 multicenter Netherland randomized controlled trail (RCT) by Daniels, et al. investigated observational versus antibiotic treatment for the first episode of CT-proven uncomplicated acute diverticulitis. This study involved 528 patients, and only Hinchey Stages 1a-b and Ambrosetti’s ‘mild’ diverticulitis stage confirmed within 24 hours by CT were included. Patients with previous diverticulitis, higher Hinchey stages, or ‘severe’ diverticulitis on Ambrosetti’s classification were excluded. The antibiotic treatment was a 10-day course of IV amoxicillin-clavulanic acid, 1,200 mg four times daily for at least 48 hours. After 48 hours, the administration route could be switched to per os (PO), 625 mg three times daily. For observational treatment, patients had to meet the criteria of tolerating a normal diet, temperature less than 100.4° F, a pain score below four on a visual analog scale (using only acetaminophen for pain control), and the ability to support self at the same level as before illness. If the patient deteriorated, CT was repeated and antibiotic treatment was started if the temperature rose above 102.2 F°, blood cultures were positive, or the patient was septic.

The primary outcome was time to recovery and was comparable for both groups: 12 days for the antibiotic treatment group and 14 days for the observational treatment group. The observational treatment group had a hazard ratio for full recovery of 0.91 when compared to the antibiotic treatment group, with no significant difference between the groups. Also, both groups had a similar percentage of patients that fulfilled the recovery criteria: 248 (93.2%) in the antibiotic group, and 234 patients (89.3%) in the observation group, with no significant difference between groups.

Secondary outcomes, such as readmission rates, showed no significant difference and were comparable: 12.0% in the antibiotic group and 17.6% in the observation group. The complicated diverticulitis, ongoing diverticulitis within six months, and recurrent diverticulitis rates were all comparable without a significant difference. No significant difference was found in the mortality rate: 0.4% for antibiotic treatment group and 1.1% for the observational treatment group. The rate of antibiotic-related adverse events was greater in the antibiotic treatment group - 8.3% compared to 0.4% in the observational group (P = 0.006) [15].

The authors found that antibiotics can be withheld from treating AUD in patients with the first episode of diverticulitis consistent with Hinchey 1a classification. This approach decreases the likelihood of antibiotic-related adverse events and does not show to be inferior to the antibiotic treatment approach when comparing time to recovery and potential complications.

Brochmann, et al. performed a single-center cohort study implementing and evaluating a non-antibiotic management protocol for acute uncomplicated left-sided diverticulitis in a large teaching hospital. All patients with CT-verified acute uncomplicated left-sided diverticulitis were admitted for observation and supportive care. Patients that were receiving antibiotics at admission, pregnant, immunocompromised, or had definite signs of severe, complicated disease were exempted and treated with antibiotics. All admissions to the hospital during the study period with an International Classification of Disease (ICD-10) code of K57.0-9 were identified retrospectively from the hospital’s electronic patient records, and investigators reviewed CT reports and discharge summaries. Patient characteristics and inflammatory parameters were recorded.

Management failures, complications, and recurrences were defined and reported. A review of all patient charts identified 244 admissions for CT-verified acute uncomplicated diverticulitis. One hundred and ninety-seven (81%) adhered to the new treatment policy of observation and supportive care. Sixty-seven patients received antibiotics on admission, and 20 of these were per protocol as they satisfied exclusion criteria. The remaining 47 admissions received antibiotics and were considered a policy violation. These two groups were not comparable, and the policy violation group tended to have a more severe clinical picture.

A retrospective analysis showed that of the 177 patients in the non-antibiotic group, 172 (97.1%) were managed successfully; five (2.8%) patients deteriorated clinically and required antibiotics, and two (1.1%) patients were readmitted due to persisting symptoms, managed without antibiotics, and did not develop any additional complications. Within a month, 173 (99.4%) patients had no complications, one (0.6%) patient developed a fistula, and three patients were lost to follow-up. At the 12-month follow-up, seven (4.6%) patients had recurrences and two (1.1%) had an elective colonic resection. Of the antibiotic treatment group, no patient experienced complications within a month; five patients (10.9%) had recurrences and one (2.2%) had an elective operation within a year. Among the patients who satisfied exclusion criteria and received antibiotics, one patient (5%) experienced a management failure and the complication rate was 30%. At follow-up, two patients (10%) had a recurrence, of whom one had a perforation and required emergent sigmoid resection [16].

The study concluded that non-antibiotic management of AUD is safe and feasible, and antibiotic treatment can be reserved for high-risk, easily-identifiable patients. As this was an observational cohort study with a retrospective analysis, it is inherently less reliable than evidence from the randomized controlled trial and cause and effect cannot be established. However, given the large success rate in those treated without antibiotics, this study still adds to the growing body of evidence that antibiotics may not be necessary for AUD.

In 2015, Isacson, et al. drafted the first study that looked at outpatient treatment for AUD without antibiotics. The study was a prospective observational study in which patients from the emergency department of two Swedish hospitals, Vastmanland and Mora, were recruited from the time period of March 2012 - December 2013 and May 2012 - August 2012, respectively. Those patients who were deemed eligible according to the inclusion criteria had a CT scan with IV contrast to confirm the diagnosis. Patients who met the diagnosis of AUD were then contacted by a nurse who assessed a daily pain score, body temperature, oral intake of food and drinks, bowel habits, and use of analgesics. They then followed up with a surgeon at one week and three months. Readmission within one month was defined as a management failure. One hundred and fifty-five patients were included in the study, 101 women and 54 men with a mean age of 57.4 years old. For 74% of the study population, it was the first episode of diverticulitis. After one week, the C-reactive protein (CRP) and the white count had normalized in 84% of the patients. A mean pain score, reported at three days, was 1.8 and 30% of patients used analgesics. Four patients failed management (2.6%) and were admitted within 14 days after being discharged from the ED while 151 patients (97.4%) were successfully managed as outpatients. Of the four patients who failed management, three had complications, two with signs of perforation and one with an abscess that was overlooked at initial presentation. The patient with the abscess was successfully treated with ultrasound-guided drainage. The fourth patient had no identifiable signs on CT [17]. These results are compared to a re-admission rate of 2.5% reported in the literature for AUD patients treated with antibiotics in the outpatient setting [18-20]. The recurrence rate within one year was 10.3% compared to the literature estimating between a 5-20% recurrence occurring in within five years [21].

Although this study is not a controlled study, when comparing the results of outpatient observation to outpatient treatment with antibiotics, readmission rates are comparable. This study strengthens previous studies that have challenged the current guidelines for the treatment of AUD [14]. Also, considering that the majority of cases of diverticulitis are uncomplicated and are admitted to the hospital, this study offers reassurance that patients with AUD can be safely treated in an outpatient setting without antibiotics with no additional risk of an adverse outcome [22-23]. Furthermore, this evidence provides incentive, given the low risk of a poor outcome, to decrease admission rates for patients with AUD, thereby decreasing the overall healthcare burden imposed by this ailment. However, caution is still advised when considering rewriting the guidelines based on this study and the prior studies. Additional research needs to be conducted to determine if the recurrence rate is comparable at the five-year mark in those treated as an outpatient with antibiotics compared to those observed as an outpatient.

Based on the results published by the AVOD study group, Isacson, et al. implemented a no-antibiotic policy at their hospital for the treatment of AUD, and the authors performed a retrospective review to see whether an increase in complications or recurrence of diverticulitis would be observed [24]. This study included 246 patients with diverticulitis of which 195 (79%) had AUD confirmed by CT. Of these, 182 patients were admitted to the hospital while 13 patients were treated as an outpatient. Of the patients that were admitted to the hospital, 165 (91%) patients were treated without antibiotics and 17 (9%) were treated with antibiotics. The 13 patients that were treated as outpatients did not receive antibiotics and had no complications.

The authors’ goal was to evaluate the complication rates and recurrence of AUD between the group treated with antibiotics and those treated without antibiotics. Recurrence was defined as a readmission after one month. Of the 165 patients treated without antibiotics as inpatients, six (4%) required readmission while one (6%) out of the 17 patients that were treated with antibiotics required readmission [24].

Although these results support the authors’ previous claim that antibiotics have no benefit in the treatment of AUD, it is important to note that the number of patients receiving antibiotic treatment for AUD was much smaller than the number of patients being treated without antibiotics. The readmission rates appear to be similar between both groups and are not statistically significant. It is also important to note that the hospital guidelines were implemented in Sweden; therefore, care must be taken when applying similar guidelines in different countries of the world, as it has been shown that diverticulitis may have regional differences in regards to severity, complications, and recurrence rates [25].

In 2011, de-Korte, et al. published a retrospective case-controlled study that evaluated the outcomes of patients with CT/ultrasound-confirmed mild AUD treated with antibiotics and without antibiotics at two different hospitals. The study was conducted in the Netherlands between January 2001 and December 2007. An arbitrary decision was made by physicians to either treat with antibiotics or observe the patient. Patients in both groups were initially given a clear liquid diet once symptoms resided and their diet was advanced as tolerated. The need for urgent or emergent surgery and/or the need for percutaneous drainage of abscesses because of clinical deterioration was considered treatment failure. The addition of antibiotics in patients initially not treated with antibiotics was not considered treatment failure but was noted. Of the 272 patients with imaging-confirmed diverticulitis, 191 were observed while 81 were treated with antibiotics. The groups did not statistically differ in age, sex, comorbidities, corticosteroid use, white blood cell (WBC) count on admission, CRP on admission, or NSAID and aspirin use. However, the two groups did differ in that a higher percentage of patients treated with antibiotics did have a higher temperature on admission (P = 0.014). There was no statistically significant difference in treatment failure between the two groups and only two patients were given antibiotics after the initial decision to place the patients in the no antibiotic group. Comparing both groups, the antibiotic group had a higher risk of recurrence, although not statistically significant [26].

Although the outcome of this study is commensurate with other literature on the subject, it does have limitations. Participants in the study were not randomized, which exposes the study to the potential for selection bias. The higher CRP and white blood cell counts in the treatment group might be an indicator of selection bias influencing this study. Nevertheless, given the high success rate in the no antibiotic group, the numbers needed to treat with antibiotics to prevent one treatment failure would undoubtedly be very high. This study was the first to show an association of non-steroidal anti-inflammatory drugs with the recurrence of diverticular disease (P = 0.037). Again, caution should be taken using this study to change clinical practice, given the difference in anatomic disease location in various geographic locations around the world [25].

## Conclusions

Diverticular disease is a common condition that places a considerable burden on the United States healthcare system. Its management not only has a significant economic impact as far as cost but also requires substantial utilization of healthcare resources. Equally concerning is the overlapping issue of antibiotic resistance, as efforts to ensure their appropriate use and reduce emerging resistance always warrant attention. In this survey of current literature, no clear evidence was demonstrated to suggest that antibiotics are requisite in the management of acute uncomplicated diverticular disease. This sharply contrasts with management guidelines based largely on expert opinion. The studies reviewed in this article provide convincing evidence that management of diverticular disease deserves reevaluation. Further, we believe these articles add to the literature by underscoring antibiotic stewardship and bringing substantial opposition to the long-seated practice strategies of managing AUD. Although the above studies have shown there is limited evidence that antibiotics should be routinely administered to patients with uncomplicated diverticulitis, it is understood that disease severity varies in uncomplicated diverticulitis, and tools are needed to better risk-stratify these patients in order to determine the appropriate treatment course. These studies have given insight into managing AUD, but we have not discounted the geographical difference between regions and believe that blinded randomized control trials are necessary in the United States in order to have any significant impact on the current management of the disease. 
